# Changes in Bacterial Population of Gastrointestinal Tract of Weaned Pigs Fed with Different Additives

**DOI:** 10.1155/2014/269402

**Published:** 2014-01-19

**Authors:** Mercè Roca, Miquel Nofrarías, Natàlia Majó, Ana María Pérez de Rozas, Joaquim Segalés, Marisol Castillo, Susana María Martín-Orúe, Anna Espinal, Joan Pujols, Ignacio Badiola

**Affiliations:** ^1^Centre de Recerca en Sanitat Animal (CReSA), UAB-IRTA, Campus de la Universitat Autònoma de Barcelona, Bellaterra, 08193 Barcelona, Spain; ^2^Departament de Sanitat i Anatomia Animals, Universitat Autònoma de Barcelona, Bellaterra, 08193 Barcelona, Spain; ^3^Institut de Recerca i Tecnologia Agroalimentaria (IRTA), Caldes de Montbui, 08140 Barcelona, Spain; ^4^Departament de Ciència Animal i dels Aliments, Universitat Autònoma de Barcelona, Bellaterra, 08193 Barcelona, Spain; ^5^Servei d'Estadística (SEA), Edifici D, Universitat Autònoma de Barcelona, Bellaterra, 08193 Barcelona, Spain

## Abstract

This study aimed to provide novel insights into the gastrointestinal microbial diversity from different gastrointestinal locations in weaning piglets using PCR-restriction fragment length polymorphism (PCR-RFLP). Additionally, the effect of different feed additives was analyzed. Thirty-two piglets were fed with four different diets: a control group and three enriched diets, with avilamycin, sodium butyrate, and a plant extract mixture. Digesta samples were collected from eight different gastrointestinal segments of each animal and the bacterial population was analysed by a PCR-RFLP technique that uses 16S rDNA gene sequences. Bacterial diversity was assessed by calculating the number of bands and the Shannon-Weaver index. Dendrograms were constructed to estimate the similarity of bacterial populations. A higher bacterial diversity was detected in large intestine compared to small intestine. Among diets, the most relevant microbial diversity differences were found between sodium butyrate and plant extract mixture. Proximal jejunum, ileum, and proximal colon were identified as those segments that could be representative of microbial diversity in pig gut. Results indicate that PCR-RFLP technique allowed detecting modifications on the gastrointestinal microbial ecology in pigs fed with different additives, such as increased biodiversity by sodium butyrate in feed.

## 1. Introduction

The weaning period is one of the most critical stages in the life cycle of pigs. This is due to the fact that, in this short period of time, the animal is yielding to many changes, such as social, environmental, and dietary changes. The combination of these news circumstances generally results in a distressing stage for the animals, favoring the occurrence of different bacterial and viral illnesses [1]. For many years, antimicrobial growth promoters (AGPs) have been used to improve the body weight gain and to control the overgrowth of opportunistic pathogens, especially during the weaning period [2, 3]. Nowadays, the increasing risk of antimicrobial resistance has led the European Union to ban all the AGPs used in animal nutrition. As a consequence, the interest in pig gut microbiota has increased considerably during the last few years. A better knowledge of the gastrointestinal bacterial composition would help to find new feed strategies to avoid pathologic infections and thus keep pigs healthier. This necessity to deepen in the knowledge of the ecosystem of the digestive tract has coincided with the appearance and implementation of molecular techniques in different fields of science and also in the microbiology that allows to have a much wider vision of these complex systems.

Currently, different additives such as plant extracts or acidifiers have been used as alternative to AGPs. The wide antimicrobial spectrum of some plant extracts such as thyme, oregano, and cinnamon has been clearly demonstrated in different *in vitro *studies [4, 5]. However, less consistent antimicrobial effects have been observed when these compounds are used *in vivo*. In chickens, a reduction of *Escherichia coli* and *Clostridium perfringens* in cecum was detected using a plant extract blend from capsicum, cinnamaldehyde, and carvacrol [6]. In weaned piglets, Manzanilla et al. and Castillo et al. [7, 8] using a similar composition mixture detected an increase of *Lactobacillus *spp. by culture and quantitative PCR, respectively. Even so, more information on the precise *in vivo* action of these compounds and their effect on microbial communities are needed to consider plant extracts as a real alternative to AGPs.

On the other hand, dietary acidification with organic acids or their salts constitutes another alternative to AGPs due to their beneficial effects on protein digestion and performance [9, 10]. It is generally accepted that the antimicrobial action of the organic acids is mainly due to the acidification of the gastric medium and to the ability of these acids to dissociate into the microbial cells, causing a shift to particular bacterial groups [11, 12]. It has been hypothesized that sodium butyrate could help to maintain the epithelium integrity, protecting the animals against pathogenic agents [13]. Moreover, Gálfi and Bokori [14] reported that sodium-butyrate supplementation in piglets reduced coliform bacteria and increased the number of *Lactobacillus* spp. in the ileum. In poultry, this supplementation also reduced coliform bacteria such as *E. coli* and *Salmonella* spp. [15]. However, few studies are available to elucidate the effect of acids on the bacterial ecosystem in the gastrointestinal tract.

Despite the general use of AGPs, their exact mode of action is not clear, and different mechanisms have been proposed. Most of them are based on the reduction of intestinal microbial mass, resulting in a decreased production of growth of depressing microbial metabolites and in the competition for nutrients with the host [16, 17]. However, other mechanisms related to the selection of a healthier microbial community could also be implicated.

The present work was designed to evaluate the existence of differences in the microbial diversity of different gastrointestinal locations by PCR-restriction fragment length polymorphism (PCR-RFLP) in weaning piglets. Moreover, a second objective was to elucidate the effect of different additives (acidifier, plant extract mixture, and antibiotic) on the gastrointestinal microbial populations.

## 2. Materials and Methods

### 2.1. Animals, Housing, and Management

The trial was performed at the Experimental Unit of the Universitat Autònoma de Barcelona and received prior approval from the University Ethical Committee for Animal Experimentation. All the procedures involving animals were conducted in accordance with the European Union Guidelines for Animal Welfare (Directiva 86/609/CEE).

Thirty-two crossbred (Pietrain × [Landrace × Large White]) mixed male and female weaned pigs were selected from ten different litters of the same farm. The pigs were weaned at 20 ± 2 days of age with an average initial body weight of 5.9 ± 0.7 kg. The piglets were distributed in 4 groups (8 animals per diet; 2 pens per diet) according to their initial weight and litter. All pigs were allocated in an environmentally controlled room and the temperature was gradually reduced from 29 to 25°C over a period of 3 weeks.

### 2.2. Diets and Experimental Design

The pigs were fed with four different experimental diets. One group (CT group) received a control diet, which was formulated, with 60% cereals, 20% milk by-products, 6% soy protein concentrate, 5% low temperature fish meal, 4% soy bean meal 44, and 4% full fat soy as the main ingredients (Table 1). The remaining three groups received the same diet where three different additives were added as follows: 0.04% of avilamycin (AB group), 0.3% of sodium butyrate (AC group) or 0.03% of a plant extract mixture standardized in 5% (wt/wt) carvacrol (*Origanum *spp.), 3% cinnamaldehyde (*Cinnamomum *spp.), and 2% capsicum oleoresin (*Capsicum annum*) (XT group). The animals were fed *ad libitum *during three weeks and had free access to water.

### 2.3. Controls and Sampling

Individual body weights (BW) were registered weekly and the average daily gain was calculated. After three weeks with the experimental diets, pigs were euthanized with an intravenous injection of sodium pentobarbital (Dolethal, Vetoquinol, S.A., Madrid, Spain) (200 mg/kg BW). The euthanasia was carried out in 2 days (day 19 and day 21 of the study) with 4 animals of each group per day.

The abdomen was immediately opened and the whole gastrointestinal tract was removed. Luminal content of all the animals was collected from stomach (ST), proximal jejunum (PJ), distal jejunum (DJ), ileum (I), cecum (C), proximal colon (PC), distal colon (DC), and rectum (R). One g of digest sample was placed in 3 mL of 98% grade ethanol and kept at 4°C until DNA extraction as described in Castillo et al. [18] and Murphy et al. [19].

### 2.4. Sample Processing

#### 2.4.1. DNA Extraction and PCR

A sample of 400 mg from the ethanol-treated digest content was washed twice with sterile buffered peptone water. DNA was extracted using the QIAamp DNA Stool Minikit (Qiagen Inc., Chatsworth, California) following the manufacturer's instructions with a minor modification, which consisted in adding 140 *μ*L of 10 mg/mL of lysozyme in Tris-EDTA buffer (pH 8.0) (Sigma Chemical Co., St. Louis, MO, USA) to the lysis buffer and incubating it at 37°C during 30 minutes to improve the DNA extraction of Gram-positive bacteria. After elution from the column, 2 *μ*L of Ribonuclease-A (Sigma Chemical Co., St. Louis, MO, USA) was added to eliminate residual RNA. Also, 4 *μ*L of 0.8 *μ*g/mL BSA (Sigma Chemical Co., St. Louis, MO, USA) was added to each sample. The DNA was stored at −20°C until PCR amplification.

PCR was performed with AmpliTaq Gold PCR-Master mix (Applied Biosystems, CA, USA) in a total reaction volume of 50 *μ*L. The PCR mix consisted of 0.05 U/*μ*L of AmpliTaq Gold polymerase, 0.8 *μ*M per each primer, 0.1% of tween 20, 5 *μ*L of template DNA (~100 ng), and autoclaved nanopure water. The 16S rDNA was amplified using the eubacterial primers: 357fm (5′-CTACGGGAGGCAGCAGT-3′) designed by Muyzer et al. [20] and 907 rm (5′-CCGTCWATTCMTTTGAGTTT-3′) by Muyzer et al. [21]. The optimized conditions for amplification were as follows: activation of TaqGold at 94°C (4 min), 35 cycles of denaturation at 94°C (1 min), annealing at 45°C (1 min) with an increment of 0.1°C per cycle, extension at 72°C (1 min 15 s), and a final extension at 72°C (15 min).

#### 2.4.2. Restriction Fragment Length Polymorphism (RFLP)

The PCR products were digested with four restriction enzymes (*AluI*, *RsaI*, *HpaII*, and *CfoI*) in four independent reactions (F. Hoffmann-LaRoche Ltd Group, Basel, Switzerland). A reaction mixture was made containing 8 *μ*L of the PCR product, 1 *μ*L SA buffer (F. Hoffmann-La Roche Ltd Group, Basel, Switzerland), and 1 *μ*L (10 U) of each restriction enzyme. Samples were incubated for 3 hours at 37°C. The different fragments were separated using a 2% high resolution agarose gel (Sigma Chemical Co., St. Louis, MO, USA) and visualized by staining with 0.5 *μ*g/mL ethidium bromide. Step ladder 50 bp (Sigma Chemical Co., St. Louis, MO, USA) was used as a DNA molecular weight marker and a mixture of PCR-RFLP from *Pasteurella multocida*, *Enterococcus faecalis*, and *Clostridium perfringens *digested with *RsaI* was processed and used as internal control. DNA bands were visualized in a UV Chemigenius Image System (SynGene, Cambrige, UK) using the GeneSnap software (SynGene Analysis Cambridge, UK, version 3.02.00), and the size of the restriction fragments was calculated with the Gene Tools software version 3.02.00 (SynGene Analysis Cambridge, UK). To reduce subjective variation during gel observation, peaks with an intensity under 60 units were discarded. Background noise was eliminated using the 30-radium rolling disk method. Finally, four band profiles for each sample were obtained which corresponded to the digesting with the four aforementioned restriction enzymes.

### 2.5. Analysis of Band Patterns

With the analysis of the restriction fragments, microbial diversity and the similarity degree were calculated. To calculate the microbial diversity two parameters were used: the total number of bands and the Shannon-Weaver *H*′ index [22]. The total number of bands was calculated for each of the samples collected in the study as a total sum of the bands obtained in the four enzymatic reactions. The Shannon-Weaver index (*H*′) was calculated using digesta samples from the distal jejunum and proximal colon by means of the following function: *H*′ = −∑*Pi*log*Pi*, where *Pi* was the importance probability of finding a given band in a tract. *Pi* was itself calculated with the function *Pi* = *ni*/*nt*, where *ni* is the height in the densitometric curves (intensities of the bands) of a given peak and *nt* is the total sum of all the peaks of a densitometric curve. The final value of the Shannon-Weaver index was obtained as the average of the Shannon-Weaver calculated for each animal.

To compare the similarity in the bacterial composition of the different experimental diets and intestinal tracts, several dendrograms were built by calculating the Manhattan distance (MD) [23]:

(1) $$\begin{array}{c} {\text{MD}}_{\left(\text{SA},\;\text{SB}\right)}=100-\frac{\sum_{\text{IP}}^{\text{FP}}\sum_{\text{IE}}^{\text{FE}}\left|\text{ISA}\left(\text{nP},\text{mE}\right)-\text{ISB}\left(\text{nP},\text{mE}\right)\right|}{\left(\text{FP}-\text{IP}\right)*\left(\text{FE}-\text{IE}\right)}, \end{array}$$

where MD (SA, SB) is the Manhattan distance between the electrophoretic profiles of sample A and sample B, IP is initial electrophoretic position, FP is final electrophoretic position, IE is initial restriction enzyme, FE is final restriction enzyme, ISA (nP, mE) is intensity of electrophoretic profile of sample A at the n position of m restriction enzyme, and ISB (nP, mE) is intensity of electrophoretic profile of sample B at the n position of m restriction enzyme.

The values of this coefficient range from 0 to 100. Both the presence and the absence of a band and its intensity (peak height of densitometric curves) were taken into account to calculate them. Using the distance matrix, different dendrograms were created using the neighbour-joining algorithm.

### 2.6. Statistical Analysis

The effect of the diet and the gastrointestinal tract on the number of bands was examined through nonparametric tests (Kruskal-Wallis) using the SAS statistical package (SAS Institute, INC. 8.2, Cary, NC). The statistically significant results (*P* < 0.05) were later administered by the Wilcoxon test (2 × 2 comparisons) applying the Bonferroni correction for multiple comparisons. The effects of the diet on the Shannon-Weaver index and on the productive parameters were analyzed by means of the SAS GLM procedure (general linear model). For all the analyses, statistical significance was determined for values of *P* < 0.05.

## 3. Results

### 3.1. Clinical and Production Parameters

No clinical signs and no diarrhoea episodes were observed in any animal during the whole experimental period. There were no significant differences in growth performance (*P* > 0.05). However, the animals that received diets with some additive had a tendency to have a higher average daily weight gain than the CT group (124.7 ± 19.1, 177.4 ± 57.5, 177.6 ± 33.1, and 165.9 ± 37.4 g for CT, AB, AC, and XT, resp., *P* = 0.069).

### 3.2. Microbial Diversity

#### 3.2.1. Number of Bands

**Figure 1:**
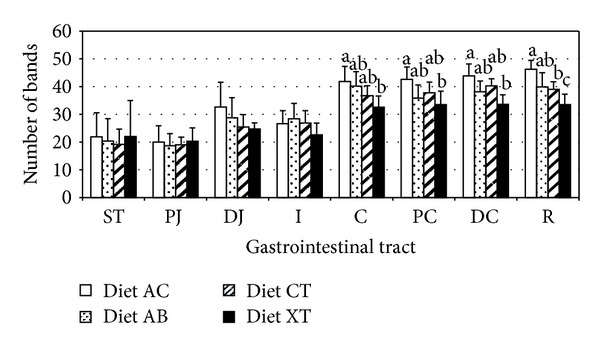
Microbial diversity in the different gastrointestinal segments. The pigs were fed with a control diet (CT) or with the same diet with avilamycin (AB), sodium butyrate (AC), or with a mixture of plant extracts (XT). The samples were collected from digestive contents in the stomach (ST), the proximal jejunum (PJ), the distal jejunum (DJ), the ileum (I), the proximal colon (PC), in the distal colon (DC), and the rectum (R). Error bars stand for standard deviation. Different letters (a, b, c) show significant differences among treatments for the same tract (*P* < 0.05).

The number of the bands from each gastrointestinal section and for each animal resulted from the total sum of the four enzymatic restrictions. The average value of the number of bands ranged from 18 to 46 per group (Figure 1). For all the diets, the number of bands was higher in the distal intestinal tracts (with values between 32.5 and 46.9) than in the proximal intestinal tracts (from 18.6 to 32.62) (*P* < 0.05). This effect was more remarkable in the AC and AB diets.

In some sections of the gastrointestinal tract (specifically in the distal jejunum and in the cecum) of the animals fed with the same diet, significant differences were observed among the four animals sacrificed on day 19 and the four sacrificed on day 21. This “sacrifice effect” was observed in the two aforementioned specific tracts in all the experimental diets. In the distal jejunum, animals from group AC euthanized on day 19 showed an average of 38 bands, whereas the animals euthanized on day 21 had an average of 27 bands (*P* = 0.0029). In the cecum, the four animals of group AC sacrificed on day 19 showed an average of 45 bands while the ones that were sacrificed on day 21 had an average of 38 bands (*P* = 0.02).

Statistically significant differences among the diets were basically observed in the large intestine (C, PC, DC, and R) (Figure 1). The most significant differences were observed between the AC and XT diets. A higher number of bands was observed in the animals fed with the AC diet and lower numbers in those fed with the XT diet (Figure 1).

#### 3.2.2. Shannon-Weaver Index

The Shannon-Weaver index (*H*′) was calculated exclusively using digesta samples from the distal jejunum and proximal colon. In all experimental diets, the degree of diversity measured by Shannon-Weaver index was lower in distal jejunum than in proximal colon (Table 2) (*P* = 0.0001). In the jejunum, numerical but not statistically significant differences were detected among experimental diets (Table 2). However, a decrease of the microbial diversity was observed from distal jejunum samples from the first day of sacrifice (*H*′ = 1.32) to the second day of sacrifice (*H*′ = 1.20), regardless of the experimental diet.

In proximal colon, the microbial diversity was affected by the composition of the diet (Table 2). Animals fed with the AC diet had a higher microbial diversity than others fed with other diets. In addition, animals fed with XT diets had a lower microbial diversity than those fed with CT diets too. No statistically significant differences were observed between the microbial diversity of the animals fed with AB diet and the animals fed with the CT diet.

### 3.3. Similarity in the Bacterial Composition

**Figure 2:**
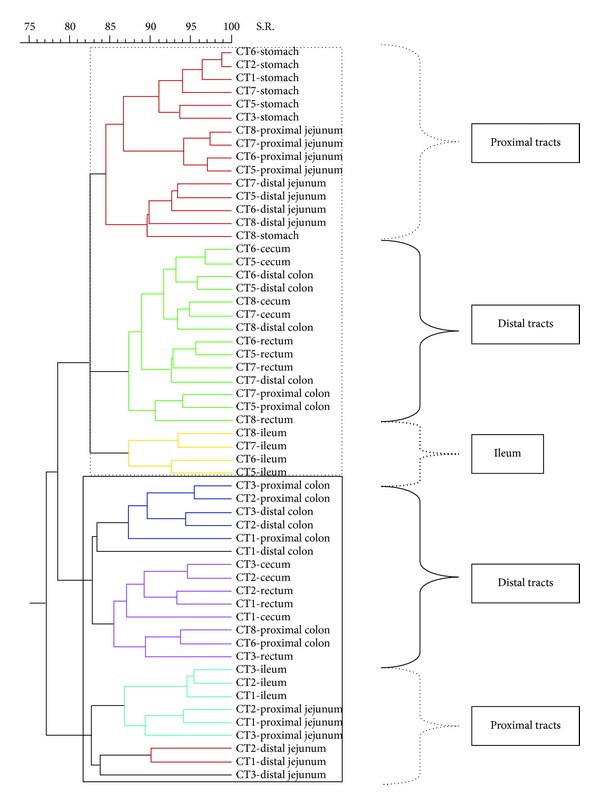
Dendrogram illustrating the similarity among band patterns obtained with PCR-RFLP among different gastrointestinal segments in pigs fed with a control diet (CT). Under the curly bracket represented with discontinuous line, the samples of proximal intestinal tracts are grouped; under the curly bracket with solid line the posterior intestinal tracts are clustered and under the curly bracket with thick discontinuous line the samples from the ileum tract are clustered. Inside the discontinuous line box the animals euthanized on day 21 are clustered and inside the continuous line box the animals euthanized on day 19 are clustered. The identification of each pig is shown in each sample.

**Figure 3:**
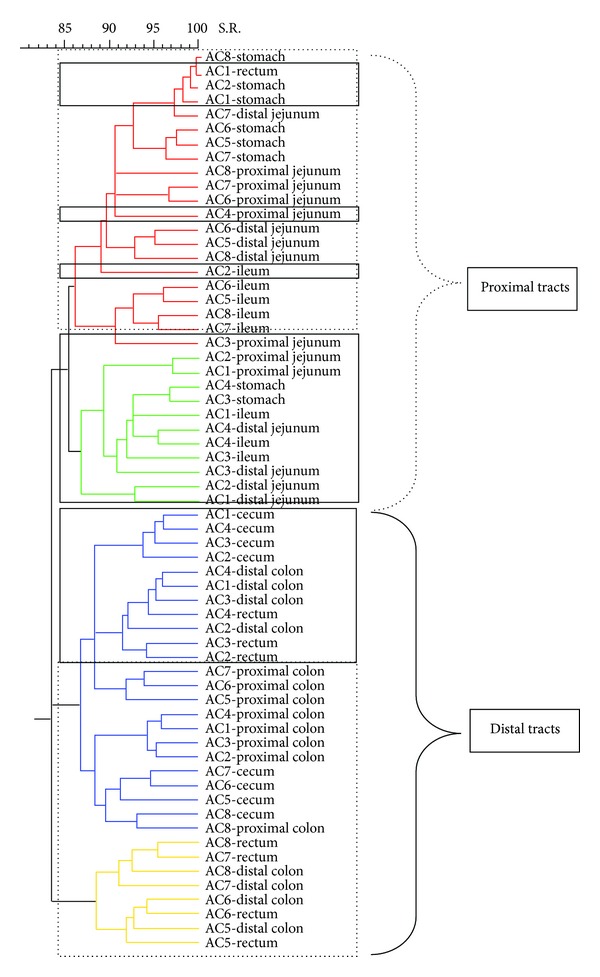
Dendrogram illustrating the similarity among band patterns obtained with PCR-RFLP among different gastrointestinal segments in pigs fed with a diet with sodium butyrate (AC). Under the curly bracket represented with discontinuous line, the samples of proximal intestinal tracts are grouped; under the curly bracket with solid line the posterior intestinal tracts are clustered. Inside the discontinuous line box the animals euthanized on day 21 are clustered and inside the continuous line box the animals euthanized on day 19 are clustered. The identification of each pig is shown in each sample.

**Figure 4:**
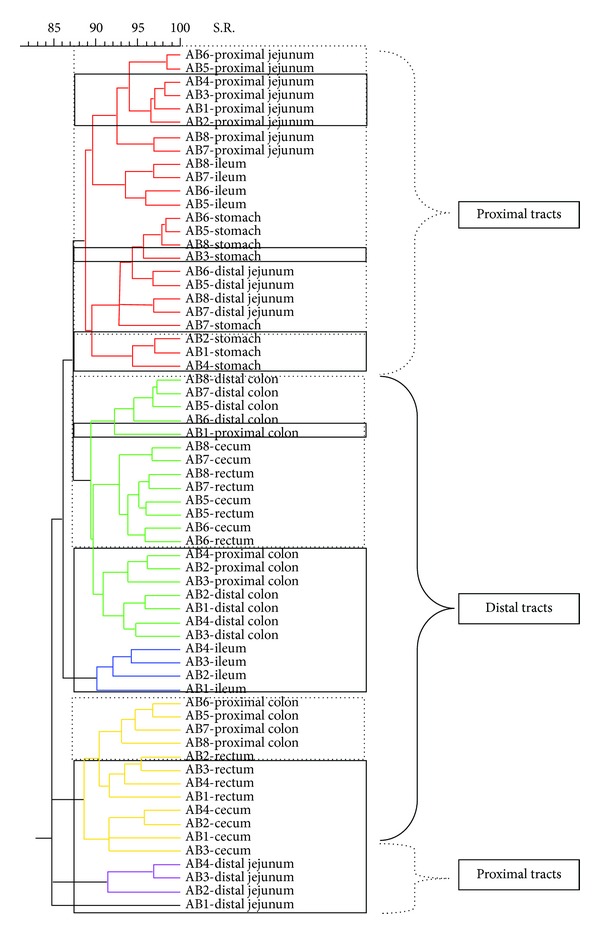
Dendrogram illustrating the similarity among band patterns obtained with PCR-RFLP among different gastrointestinal segments in pigs fed with a diet with avilamycin (AB). Under the curly bracket represented with discontinuous line, the samples of proximal intestinal tracts are grouped; under the curly bracket with solid line the posterior intestinal tracts are clustered. Inside the discontinuous line box the animals euthanized on day 21 are clustered and inside the continuous line box the animals euthanized on day 19 are clustered. The identification of each pig is shown in each sample.

**Figure 5:**
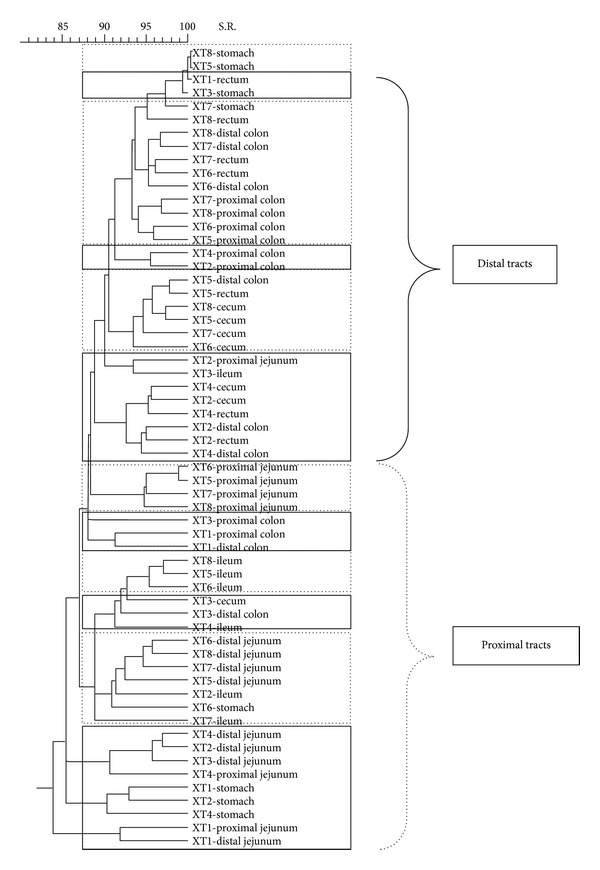
Dendrogram illustrating the similarity among band patterns obtained with PCR-RFLP among different gastrointestinal segments in pigs fed with a diet with a mixture of plant extracts (XT). Under the curly bracket represented with discontinuous line, the samples of proximal intestinal tracts are grouped; under the curly bracket with solid line the posterior intestinal tracts are clustered. Inside the discontinuous line box the animals euthanized on day 21 are clustered and inside the continuous line box the animals euthanized on day 19 are clustered. The identification of each pig is shown in each sample.

**Figure 6:**
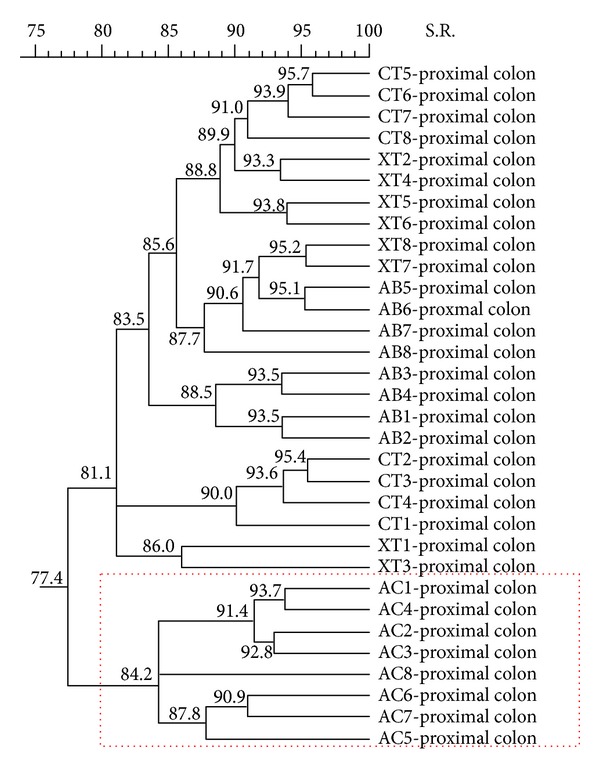
Ecological changes in microbial populations in proximal colon. Dendrogram illustrating the similarity among band patterns from proximal colon digest samples when comparing the four different experimental diets in weaning piglets. The experimental diets are indicated as control diet (CT) or the same diet with 0.04% avilamycin (AB), 0.3% sodium butyrate (AC), or 0.03% plant extract mixture (XT). The pig identification numbers are indicated for each sample.

**Figure 7:**
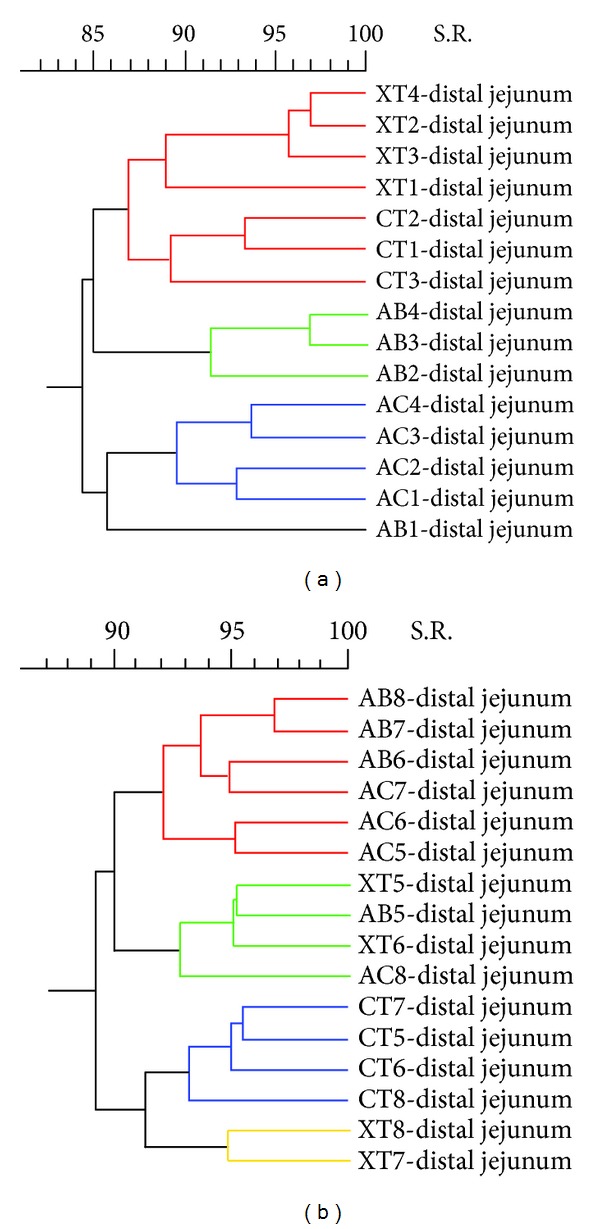
Ecological changes in microbial population in distal jejunum. Dendrograms show the percentage of similarity of the band patterns when comparing the four experimental diets for the animals sacrificed on day 19 (a) and for the animals sacrificed on day 21 (b). The experimental diets are indicated as control diet (CT) or the same diet with 0.04% avilamycin (AB), 0.3% sodium butyrate (AC), or 0.03% plant extract mixture (XT). The pig identification numbers are indicated for each sample.

The similarity of bacterial populations between different intestinal tracts and different diets was evaluated with dendrograms which were built by calculating the Manhattan distance (Figures 2 to 7). The dendrogram formed with the band pattern of the different gastrointestinal tracts of pigs fed with a CT diet was shown in Figure 2. In this dendrogram, 2 clusters were observed depending on the day of sacrifice. The animals euthanized on day 19 (numeration from 1 to 4) are clustered in one branch, whereas the animals euthanized on day 21 (numeration from 5 to 8) are clustered in another branch. This sacrifice effect was not observed for the stomach samples. However, this effect was less clear in groups of animals fed with AC, AB, and XT diets (Figures 3, 4, and 5, resp.).

Analysing with more detail the different branches of the dendrogram of the animals fed with CT diets, it was possible to observe different clusters depending on the gastrointestinal section. Inside the group of animals sacrificed on day 19, it was possible to observe 2 subgroups. One subgroup was formed by distal intestinal segments (C, PC, DC, and R) and the other by the proximal tracts (PJ, DJ, and I). In the case of the animals euthanized on day 21, it was possible to observe 3 different clusters: one subgroup created by ST, PJ, and DJ samples, another subgroup constructed with different segments of the distal intestinal tract (C, PC, DC, and R), and a well-defined third subgroup created with the I samples. This separation between the proximal tracts and the posterior tracts was also observed in the other experimental diets (Figures 2 to 5).

To analyze the effect of the diets on a specific intestinal tract, different dendrograms were generated. We did not observe a clear cluster produced by diets on the microbial population from ST, PJ, DJ, I, C, DC, and R. Conversely, when pigs were fed with the AC diet, changes in the microbial population were detected in the proximal colon (Figure 6) and formed a distinctive cluster.

Because a sacrifice effect was previously observed in the biodiversity degree of the distal jejunum and in the cecum, dendrograms of these two intestinal tracts were generated with the animals sacrificed on day 19 and with the animals sacrificed on day 21 separately. Remarkably, in the distal jejunum, the animals euthanized on the first day showed a cluster depending on the diets, whereas the animals sacrificed on the second day did not show any specific association (Figure 7).

## 4. Discussion

The microbial population of the gastrointestinal tract of pigs has traditionally been studied by culture techniques [24, 25]. In recent years, molecular techniques have been introduced to improve the detection of bacteria, which are fastidious to culture [26, 27]. The use of these new techniques has allowed a better characterization of the composition of the intestinal microbiota.

In this work, the use of the PCR-RFLP technique permits a broader view of the composition of a complex bacterial ecosystem, such as the gastrointestinal tract of animals. In addition, the use of this technique allows us monitoring general variations that may occur in a bacterial population due to a change of the diet, time, and so forth. Also, with the PCR-RFLP technique we are exploring either unculturable and culturable bacteria or uncharacterized bacteria. It is estimated that the microbial community of the colon is composed of 400–500 different bacterial species [28]. A broader view of the ecosystem of the digestive tract can be obtained by PCR-RFLP. Although the number of resulting bands is relatively high (maximum 46), it cannot be ruled out that this technique might be underestimating the real degree of bacterial diversity. Such possibility may be due to a potential preferential amplification of certain bacterial species causing an insufficient amplification of the DNA of less prevalent bacteria. However, information provided by PCR-RFLP is always greater than that obtained from the cultivation of some specific bacterial groups.

In this study, using PCR-RFLP, it was possible to assess the diversity and similarity of the gastrointestinal microbial populations in relation to the different sections of the digestive tract and the different experimental diets. We can observe that microbial diversity increased significantly in more distal gastrointestinal segments than in the proximal sections. These results show a good concurrence with the results reported by Konstantinov et al. or Wang et al. [29, 30]. Several factors such as more neutral pH, slow intestinal transit, and/or low oxidation-reduction potential are associated with increased survival of bacteria in the hindgut [31]. In contrast, conditions applying to more proximal sections of the digestive tract (more acid environment, fast transit, and high bile acid concentration) make microbial diversity lower, with higher abundance of acid lactic bacteria [32]. Furthermore, when the band patterns are analyzed by creating dendrograms, proximal segments (stomach, proximal jejunum, and distal jejunum) and distal segments (cecum, proximal colon, distal colon, and rectum) show a tendency to be clustered. The ileum content samples formed a clearly distinct group, separated from both sections of the proximal bowel and distal intestinal segments. This result may be related to the fact that the ileum has some special features, such as pH being more similar to the caecum than to the contiguous small intestine. Besides, ileum has prominent Peyer's patches, a histological and immunological unique intestinal structure, which may affect its bacterial population.

Microbial diversity changed depending on the day of sacrifice in some parts of the digestive tract, especially in distal jejunum and cecum. The sacrifice effect was also observed when studying the degree of similarity in some of the dendrograms (CT diet). Besides, the animals euthanized on the first day showed a cluster of similarity depending on the diets, whereas this effect disappears on the second day of sacrifice. This effect could be related to the stress generated by the sacrifice of half of the experimental group (half of the animals in a pen on first day) and to the subsequent modification of the hierarchy of this group. It has been shown that after acute stress, bacterial populations in the digestive tract can be altered quickly. Williams et al. [33] observed a decrease in the homogeneity of the band patterns after transporting the pigs. Consequently, the sacrifice effect and any factors that could alter the behavior of the animals may be important and should be taken into account in the design of experiments that analyze intestinal microbiota.

Apart from the ecological analysis of the microbiota throughout the gastrointestinal tract, this study also describes the effect of the incorporation of avilamycin, sodium butyrate, and a plant extract mixture on the gastrointestinal microbial populations. Adding these feed additives, the most significant differences are found in distal gastrointestinal segments, especially in the group of animals fed with the diet enriched with sodium butyrate or with a mixture of plant extracts. Thus, animals fed with the AC diet have a higher microbial diversity, while animals which were fed with plant extracts have a lower bacterial diversity. Gálfi and Bokori [14] observed changes in the ileal microbiota using 0.17% sodium n-butyrate in diets for weaned piglets. They detected a decrease in the proportion of coliform bacteria with a simultaneous increase in lactobacilli. Additionally, Van Immerseel et al. [15], using microencapsulated butyric acid in young chickens, could also demonstrate a decrease colonization of *Salmonella* spp. in the caecum after an experimental infection. These two studies exhibit the evidence that butyrate acid exerts some effects on microbiota. However, there are no studies analysing the overall effect of butyrate acid on the microbial biodiversity along the gastrointestinal tracts. In the present study, an increase of microbial diversity was observed in proximal colon. The so called biodiversity has been proposed as an indicator of intestinal microbiota stability [34]. The increase of microbial diversity in proximal colon of AC pigs could be one of the factors that could explain the better numerical performance observed in these animals.

Moreover, other feed acidifiers have also demonstrated an effect on microbial diversity. Torrallardona et al. [35] detected an increased microbial diversity in the ileum using a diet with 0.5% of benzoic acid. On the other hand, Canibe et al. [36] detected a reduction of microbial diversity in both proximal intestinal segments and colon using a diet with formic acid. Factors such as the type of acid (a single acid or a mixture), dose, tolerance, and mode of action might explain these contradictory effects.

It is expected that the action of the organic acids will be higher in the proximal portions of the digestive tract (stomach and small intestine) [37]. However, in our study, the effect of sodium butyrate was observed on the bacterial ecosystem of the more distal intestinal segments rather than in the proximal portions. The increase in microbial diversity, found in animals fed with the AC diet, could be related to some direct effect (caused by a metabolite derived from the sodium butyrate) or an indirect effect (reduction of some bacterial species that, in turn, control the concentration of other bacterial species) of the sodium butyrate on bacterial populations of the proximal gastrointestinal tract. Thus, Van Winsen et al. [38] hypothesized that the number of bacteria in the Enterobacteriaceae family in the stomach determined the level of these bacteria in faeces. The authors attributed this result to an effect of the microbiota in proximal sections over the subsequent intestinal segments.

In a study performed in parallel and using the same samples as those used in this study [39], the authors found increased concentrations of butyric acid in the stomach due to its inclusion in the diet (CT = 4.87, AB = 5.11, and XT = 2.98 versus AC = 15.54, SEM = 0.970, *P* = 0.0001). However, the concentration of this acid showed no significant differences among experimental diets in other gastrointestinal segments. This could support the hypothesis that the effect of sodium butyrate on the hindgut microbiota might be an indirect effect.

The decrease in microbial diversity in animals fed with plant extracts has been previously reported in some studies [40]. This reduction in microbial diversity may be due to the antimicrobial effect of plant extracts, inhibiting some bacterial groups and promoting specific bacterial groups. In this case, in a parallel study using the same samples and the quantitative PCR technique, Castillo et al. [8] detected a significantly increased number of lactobacilli in the cecum in animals fed with the diet enriched with extracts of plants. However, in this same study, the authors found no significant differences in the quantity of total bacteria, either in animals fed with a diet enriched with sodium butyrate or in those fed with the diet enriched with plant extracts. These results indicate that these additives produce qualitative changes on the bacterial population of the gastrointestinal tract without affecting the total amount of bacteria. This fact has already been described in another study [36].

Our results show that animals fed with the control diet and animals fed with a diet enriched with avilamycin have similar microbial richness. This result is observed throughout the digestive tract. Collier et al. [41], using PCR-DGGE to study the bacterial composition in the ileum in pigs treated with tylosin for 21 days, observed a similar number of bands between the animals fed with the control diet and the animals receiving the food supplemented with the antibiotic. This outcome is attributed to an adaptation of the microbiota of antibiotic administration and may indicate a substitution of bacteria sensitive to antibiotics by bacteria resistant to them.

## 5. Conclusion

In conclusion, changes in the complexity of the bacterial populations can be detected throughout the gut and with different additives by PCR-RFLP. Microbial diversity significantly increases from the small intestine to the large intestine. Additionally, obtained results suggest that the selection of proximal jejunum, ileum, and proximal colon are the most representative intestinal segments to study microbial diversity in pig gut. Besides, feed additives (such as sodium butyrate, a plant extract mixture, or avilamycin) are able to modify the gastrointestinal microbial ecology. The inclusion of sodium butyrate in weaned piglet diets increased the microbial biodiversity in distal intestinal segments, whereas the use of a mixture of plant extracts reduced it. More specific studies are required to clarify how these changes of the microbial diversity are significant factors to achieve a successful weaning process.

## Figures and Tables

**Table 1:** Control diet composition, as fed basis.

Ingredient	g/kg
Corn	276
Barley	300
Soybean meal, 44% CP	40
Full fat extruded soybeans	40
Soya protein concentrate	60
Fish meal LT^a^	50
Dried whey	40
Acid whey^b^	150
Wheat gluten	6.8
Sepiolite	10
Dicalcium phosphate	11
L-Lysine·HCl	4.4
DL-Methionine	2.7
L-threonine	1.9
L-tryptophan	0.4
Choline chloride, 50% choline	2.0
Chromic oxide	1.5
Vitamin and mineral premix^c^	3.0
Calculated nutrient composition^d^	
GE, Mcal/kg	4.75
Crude protein, g/kg	183.9
Ether extract, g/kg	51.1
Crude fiber, g/kg	27.8
Ca, g/kg	6.44
*P* total, g/kg	6.95
*P* available, g/kg	4.01
Lysine, g/kg	13.87

^a^Fish meal low temperature: product obtained by removing most of the water and some or all of the oil from fish by heating at low temperature (<70°C) and pressing.

^b^Acid whey: product obtained by drying fresh whey (derived during the manufacture of cheeses) that has been pasteurized.

^c^Provided the following per kilogram of diet: vitamin A, 13,500 IU; vitamin D3, 2000 IU; vitamin E, 80 mg; vitamin K3, 4 mg; thiamine, 3 mg; riboflavin, 8 mg; vitamin B6, 5 mg; vitamin B12, 40 *µ*g; nicotinic acid, 40 mg; calcium pantothenate, 15 mg; folic acid, 1.3 mg; biotin, 150 *µ*g; Fe, 120 mg as iron carbonate; Cu, 175 mg as copper sulfate 5H_2_O; Zn, 110 mg as zinc oxide; Mn, 65 mg as manganese sulfate; I, 1 mg as potassium iodate; selenium, 0.10 mg as sodium selenite.

^d^Based on composition values from NRC (1998).

**Table 2:** Effect of the diet on the microbial diversity (Shannon-Weaver index) in the distal jejunum and proximal colon digesta. The pigs were fed with a control diet (CT) or with the same diet with avilamycin (AB), sodium butyrate (AC), or with a mixture of plant extracts (XT). Different letters (a, b, c) show significant differences among treatments for the same tract (*P* < 0.05).

Shannon-Weaver index	AC	AB	CT	XT	SEM*	*P* value diet	*P* value day	*P* value diet ∗ day
Distal jejunum	1.32	1.26	1.23	1.22	0.028	0.07	0.0004	0.77
Proximal colon	1.48^a^	1.39^bc^	1.42^b^	1.37^c^	0.002	0.002	0.098	0.34

*SEM: standard error of the mean.
